# Examining trends in health care access measures among low-income adult smokers in Ohio: 2012–2019

**DOI:** 10.1016/j.pmedr.2022.102106

**Published:** 2023-01-02

**Authors:** Andreas A. Teferra, Jeffrey J. Wing, Bo Lu, Wendy Xu, Megan E. Roberts, Amy K. Ferketich

**Affiliations:** a Center for Child Health Equity and Outcomes Research, Abigail Wexner Research Institute, Nationwide Children’s Hospital, Columbus, OH, USA; b Division of Epidemiology, College of Public Health, The Ohio State University, Columbus, OH, USA; c Division of Biostatistics, College of Public Health, The Ohio State University, Columbus, OH, USA; d Division of Health Services Management and Policy, College of Public Health, The Ohio State University, Columbus, OH, USA; e Division of Health Behavior and Health Promotion, College of Public Health, The Ohio State University, Columbus, OH, USA

**Keywords:** Smokers, Health care access, Medicaid, Preventive care, Low-income

## Abstract

Smokers are more likely to be low-income with limited access to health services. Although Medicaid expansion under the Patient Protection and Affordable Care Act improved access to care for low-income adults, long-term trends in health care access among low-income smokers remain uncharacterized. The study evaluated changes in five access measures among low-income nonelderly (19–64) adults (N = 28976) across smoking status using pooled data from a statewide survey in Ohio covering pre- (i.e., 2012) and post-Medicaid expansion periods (2015, 2017, and 2019) guided by a comprehensive framework of health care access. We found improvements in some, but not all, health care access measures among low-income smokers in the post-Medicaid-expansion period compared to the pre-expansion period. Compared to 2012, the odds for unmet dental care needs declined in 2015 (aOR = 0.67, 95 % CI = 0.45–1.01), 2017 (aOR = 0.53, 95 % CI = 0.35–0.81), and 2019 (aOR = 0.65, 95 % CI = 0.40–1.05) (p trend < 0.001). Similarly, the odds for unmet other health care needs (i.e., medical exams and supplies) were lower in 2015 (aOR = 0.64, 95 % CI = 0.39–1.06), 2017 (aOR = 0.56, 95 % CI = 0.34–0.93), and 2019 (aOR = 0.47, 95 % CI = 0.27–0.83) (p trend < 0.001). Difficulty paying medical bills was also significantly lower in 2015 (aOR = 0.62, 95 % CI = 0.43–0.89), 2017 (aOR = 0.57, 95 % CI = 0.39–0.83) and 2019 (aOR = 0.57, 95 % CI = 0.37–0.87) (p trend < 0.001). While there was notable progress in measures of affordability (i.e., paying medical bills) as well as care availability and accommodation (i.e., unmet needs), there were no meaningful changes in the approachability of care (i.e., having a usual source of care).

## Highlights

• Changes to access to care measures among low-income smokers following Medicaid expansion have not been characterized.
• Health care access measures among smokers related to affordability of care and unmet needs improved following Medicaid expansion in Ohio.
• The was no change in having a usual source of care, an important indicator of preventive care use.
• A comprehensive improvement on access to care is essential for tobacco cessation efforts, early management of health problems, and reduction in smoking-related health care costs and disparities.

## 1 Introduction

Although the percentage of adult smokers in the U.S. has declined by nearly 7 % from 2005 to 2019 (20.9 % vs 14.0 %), (Cornelius, 2020) the prevalence of smoking among low-income adults remains considerably high. In 2020, adults with an annual income lower than $35,000 were more than three times as likely to smoke compared to those with an income of $100,000 or higher (20.2 % vs 6.2 %), and smoking among low-income adults who were enrolled in Medicaid or were uninsured was more than double the rate of those on private insurance (22.7 % and 21.2.% vs 9.2 %, respectively). (Cornelius, 2022).

As part of the Patient Protection and Affordable Care Act (ACA), many states expanded their Medicaid coverage to low-income adults aged 19 to 64 (i.e., at or below 138 % of the federal poverty level (FPL)). As a result, between 2013 and 2017, the proportion of uninsured adults was nearly halved from 15 % to 7.8 %, (Agency for Healthcare Research and Quality, 2019) and just a year after most states expanded Medicaid coverage in January 2014, (KFF, 2022a) an estimated 2.3 million adult smokers gained health insurance coverage nationwide. (DiGiulio, 2016) Furthermore, by regarding tobacco cessation efforts as “preventive science,” (American Lung Association, 2020) Medicaid expansion increased coverage for tobacco treatment and access to counseling services by removing prior authorization and cost-sharing for smokers seeking treatment. These policy changes were associated with improved access to care among smokers in various states, (Bailey et al., 2020, Brown et al., 2018) with Medicaid expansion credited with higher cessation rates (Bailey et al., 2020, Greene et al., 2014) and reduced smoking prevalence. (Hilts et al., 2021, Valvi et al., 2019).

Most recent studies on health care services among smokers have focused on utilization (Azagba et al., 2013) or costs, (Xu et al., 2015) with little evidence on access to care, a determinant of health care use and subsequent health outcomes. (Andersen, 1995) However, it is known that cigarette smokers have lower access and face more frequent barriers to health care services than non-smokers. For example, smokers are less likely to have a usual source of care (USC) (Kiefe et al., 1998, Garg et al., 2021)—a medical professional or setting where an individual routinely seeks health services when ill or in need of health consultation. Smokers are also more likely to report unmet dental (DiGiulio, 2016, American Lung Association,2020) and mental health (Iyer et al., 2019) needs. The ability to access health care is integral to the health outcomes of smokers because of their health care needs and increased risk for various health problems. Likewise, establishing a USC determines smokers’ use of overall preventive care, early detection and treatment of smoking-specific diseases like lung cancer, (Blewett et al., 2008, Xu, 2002, Chalian et al., 2019) and changes in smoking behavior. (Kim et al., 2020, Tilert and Chen, 2015).

Although the impact of Medicaid expansion on access has been evaluated across various studies, a comprehensive assessment of long-term trends in access measures following Medicaid expansion among low-income smokers is absent. There is also no comparison of these trends with other low-income subgroups (i.e., non-smokers), which could help identify unique access determinants and barriers that might disproportionately affect smokers. Further highlighting the need for additional research among low-income and disadvantaged groups is the recent uptick in the number of uninsured adults following a period of sustained decline in the overall uninsured rate nationwide. (Tolbert et al., 2020).

Improvement of health care access such as receiving needed care, having a primary care provider, and increasing access to smoking cessation services are among the key objectives of the Healthy People 2030 framework. (Office of Disease Prevention and Health Promotion and Department of Health and Human Services, 2022) These objectives call for an enhanced understanding of health care access and its determinants for vulnerable populations such as low-income smokers. Thus, aiming to better understand changes in health care access post-Medicaid expansion in Ohio, this study assessed trends in health care access measures among smokers. As a sub-aim, this study also contrasted changes in access measures between current smokers and non-smokers (i.e., former and never smokers).

### 1.1 Theoretical framework

**Fig. 1:**
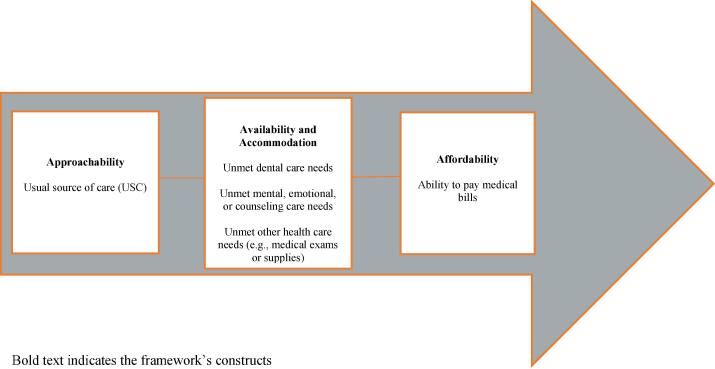
Mapping health care access measures to Levesque’s Framework of Health Care Access.

The study was guided by Levesque’s Conceptual Framework of Health Care Access (Levesque et al., 2013) which proposes a multidimensional view of health care access, presenting it as a complex interaction between user-related factors, health system factors, and various contextual characteristics. Of particular relevance to this study are the Framework’s constructs of (1) approachability, (2) availability and accommodation, and (3) affordability. Approachability is related to factors that help people identify, reach, and understand the role of health care including the information on services that are provided. Availability and accommodation include determinants of accessing and reaching care in a timely manner (e.g., availability and accessibility of facilities) while affordability relates to the economic ability to seek appropriate care and pay for services. We mapped health care access measures into components of the health system relating to these three constructs (Fig. 1).

## 2 Methods

### 2.1 Setting and participants

The Ohio Medicaid Assessment Survey (OMAS) is a statewide survey carried out in Ohio about every-two years among noninstitutionalized adults 19 years of age or older to assess and track the health status of residents over time. (Ohio Medicaid Assessment Survey | Ohio Colleges of Medicine Government Resource Center, 2021) The current study restricted the analytic sample to low-income Medicaid-eligible nonelderly adults (i.e., 138 % or lower of the FPL and between 19 and 64 years of age). We limited our sample to non-elderly adults because that is the age range, in tandem with income, used for Medicaid eligibility as part of the ACA. An additional reason for not including adults above 65 is the presence of near-universal access to care provided by Medicare and the low prevalence of smoking among seniors. (Cornelius, 2022) The study used data across four consecutive cycles of OMAS from 2012 (N = 4736), 2015 (N = 9111), 2017 (N = 8697), and 2019 (N = 6438).

### 2.2 Procedures

OMAS uses a complex, stratified, probability-based sampling design with a combination of random-digit-dialing of landline telephone numbers, random sampling of cell phone numbers, and, most recently, an address-based sampling method. (Ohio Medicaid Assessment Survey | Ohio Colleges of Medicine Government Resource Center, 2021) Eligible respondents include those who have lived in Ohio for at least one month before the interview. If a household contained more than one eligible adult, the person with the most recent birthday was selected for the interview. Proxy respondents were used for adults who could not complete the interviews. The survey used computer-assisted telephone interviewing as the primary data collection method with interviews conducted in English or Spanish. The institutional review boards at The Ohio State University and Research Triangle Institute (RTI) International approved the study protocol for OMAS.

### 2.3 Measures

*Dependent variables.* Health care access was assessed using several separate measures: 1) having no USC, 2) unmet dental care, 3) unmet mental, emotional health, or counseling care needs, 4) unmet other health care needs such as medical exams or medical supplies, and 5) difficulty paying medical bills. These measures were mapped to the dimensions of access using Levesque’s Framework of Health Care Access (Fig. 1). (Levesque et al., 2013) Participants were classified as having no USC if they responded “no” to the prompt, “Is there one place that you usually go to when sick or you need advice about your health?” and affirmed that there was “no place at all” to the follow-up confirmatory question, “Just to be sure, is it that there is no place at all that you usually go to when sick or you need advice about your health or is it that you go to more than one place?” Participants were categorized as having an unmet health care need if they answered affirmatively to the question(s), “During the past 12 months, was there a time where you needed any of [dental care], [mental or emotional care or counseling services], [other health care, such as medical exams, or medical supplies] but could not get it at that time? The 2019 survey contained an additional response choice for each of the three unmet care needs items where few participants (N = 67, N = 109, and N = 57 for dental care, mental care, and other health care needs, respectively) indicated that they did not need care. These participants were categorized as not having unmet needs. Finally, participants who answered “yes” to the question, “During the past 12 months, were there times when you had problems paying or were you unable to pay medical bills for yourself or anyone else in the family or household?” were categorized as having difficulty paying medical bills.

*Independent variable.* Smoking status was assessed using two items: 1) “Have you smoked at least 100 cigarettes in your entire life?” and 2) “Do you smoke cigarettes every day, some days, or not at all?” Participants were classified as current smokers if they responded “yes” to the first question and “every day” or “some days” to the follow-up question. Those who answered “yes” to the first question and “not at all” to the follow-up question were classified as former smokers, and those who responded “no” to the first question were considered never smokers.

*Covariates.* Sociodemographic characteristics included age (years), gender (male, female), race/ethnicity (Non-Hispanic White, Non-Hispanic Black, Hispanic, Other), educational attainment (high school or below, some college or an associate degree, college degree or higher), marital status (married vs not married), and full- or part-time employment (employed vs unemployed). Participants’ county type was classified as metropolitan, rural Appalachian, rural non-Appalachian, or suburban. OMAS defines county type in accordance with guidance from the Appalachian Regional Commission (ARC), US Census Bureau, and the Federal Office of Rural Health Policy at the Health Resources and Services Administration (HRSA). Health insurance was categorized as Medicaid, uninsured, and other (i.e., directly purchased, employer-sponsored insurance, or self-reported other insurance plans). A chronic health condition (yes vs no) was defined as having a diagnosis of one or more of the following: hypertension, heart attack, coronary artery disease, congestive heart failure, or diabetes mellitus. Self-rated health status was measured with a standard five-point scale, with responses ranging from “poor” to “excellent.” Frequent mental distress was defined as having at least 14 days in a month where a mental health condition or emotional problem restricted oneself from doing work or other usual activities. (Cree et al., 2020).

### 2.4 Statistical analyses

*Missing data.* Variables necessary for weighting procedures (age, gender, race/ethnicity, county type, and educational attainment) were pre-imputed using a weighted sequential hot-deck single imputation, (Iannacchione, 1982) with further details provided elsewhere. (Ohio Medicaid Assessment Survey | Ohio Colleges of Medicine Government Resource Center, 2021) Item non-response in the analytic sample for smoking status, access measures, and covariates such as frequent mental distress ranged from 4.2 % to 7.2 % (Supplementary Table S1). Multiple imputation with predictive mean matching was carried out for these values. Since the missingness was small, ten imputed datasets were created. (van Buuren, 2022) Results from the multiply imputed datasets were pooled using Rubin’s rules. (Rubin, 1987).

*Outcome models.* We assessed trends in health care access measures by smoking status and year using multivariable logistic regression models, adjusted for age, gender, race/ethnicity, county type, educational attainment, and frequent mental distress. Confounder identification was conducted using a directed acyclic graph (DAG) (Greenland et al., 1999) elucidating the relationship between measured and unmeasured variables of interest (Supplementary Fig. S1). All analyses accounted for the complex sampling design of OMAS and followed proper variance estimation techniques, including considerations for subgroup analysis and adjustment of sampling weights when pooling multiple years of data. (Ohio Medicaid Assessment Survey | Ohio Colleges of Medicine Government Resource Center, 2021, Lohr, 2019, CDC, 2018) A two-sided p-value <0.05 indicated statistical significance. All analyses were carried out in R (version 4.1.2). (R Core Team, 2020).

*Sensitivity analyses.* While multiple imputation remains an essential tool for handling missing data bias and improving efficiency under specific certain assumptions about the missing data mechanisms (i.e., missing at random), in the presence of data that are not missing at random (e.g., missing completely at random), complete case analysis can produce unbiased results. (van Buuren, 2022, Hughes et al., 2019, Sterne et al., 2009) Thus, we ran complete case analyses and compared the results to the ones from the multiple imputation.

## 3 Results

Among 28,976 low-income adults (mean age 43.1 years) in the pooled sample from all years (2012–2019), 11,104 (40.5 %) were current smokers. Table 1 shows that compared to former and never smokers in the pooled sample, more current smokers were without a college degree, on Medicaid, unemployed, and reported more frequent mental distress as well as fair or poor self-rated health. Having no USC was more often reported among current and never smokers than former smokers. Table 2 shows that compared to former and never smokers, current smokers reported more unmet health care needs and difficulty paying medical bills.

**Table 1:** Weighted sample characteristics of low-income adults by smoking status: Ohio Medicaid Assessment Survey, 2012–2019.

Variables	Current smokers (N = 11104)	Former smokers (N = 5023)	Never smokers (N = 11492)	Unknown smoking status (N = 1357)	Overall (N = 28976)
Age group					
19–24	949 (12.0 %)	246 (7.5 %)	2336 (27.1 %)	249 (25.7 %)	3780 (17.8 %)
25–34	2199 (26.6 %)	689 (19.5 %)	2221 (23.3 %)	318 (29.6 %)	5427 (24.1 %)
35–44	2139 (21.8 %)	782 (19.6 %)	2013 (19.2 %)	243 (17.8 %)	5177 (20.3 %)
45–54	2777 (22.1 %)	1204 (23.0 %)	2250 (16.2 %)	266 (15.3 %)	6497 (19.7 %)
55–64	3040 (17.4 %)	2102 (30.4 %)	2672 (14.3 %)	281 (11.5 %)	8095 (18.1 %)

Sex					
Male	4453 (44.3 %)	2083 (46.1 %)	4232 (41.2 %)	596 (53.9 %)	11,364 (43.5 %)
Female	6651 (55.7 %)	2940 (53.9 %)	7260 (58.8 %)	761 (46.1 %)	17,612 (56.5 %)

Race/Ethnicity^a^					
Non-Hispanic White	7398 (74.9 %)	3430 (76.8 %)	6346 (61.9 %)	119 (32.5 %)	17,293 (69.0 %)
Non-Hispanic Black	2290 (19.0 %)	914 (16.2 %)	2999 (25.1 %)	35 (6.4 %)	6238 (20.9 %)
Hispanic	437 (3.6 %)	252 (4.4 %)	983 (7.6 %)	18 (3.8 %)	1690 (5.4 %)
Other	979 (2.5 %)	427 (2.6 %)	1164 (5.5 %)	1185 (57.3 %)	3755 (4.7 %)

County type^b^					
Metropolitan	5722 (57.5 %)	2569 (55.2 %)	6690 (62.9 %)	711 (57.7 %)	15,692 (59.4 %)
Rural Appalachian	2715 (19.5 %)	1156 (19.5 %)	2108 (15.0 %)	267 (17.4 %)	6246 (17.6 %)
Rural Non-Appalachian	1471 (11.6 %)	680 (12.1 %)	1363 (10.1 %)	188 (9.8 %)	3702 (11.0 %)
Suburban	1196 (11.4 %)	618 (13.2 %)	1331 (12.0 %)	191 (15.1 %)	3336 (12.0 %)

Educational attainment					
High school or below	7405 (69.7 %)	2795 (57.9 %)	5933 (52.5 %)	694 (60.2 %)	16,827 (60.5 %)
Some college/associate d.	3096 (27.0 %)	1640 (32.9 %)	3411 (33.2 %)	407 (28.1 %)	8554 (30.5 %)
College degree or higher	603 (3.3 %)	588 (9.3 %)	2148 (14.3 %)	256 (11.7 %)	3595 (9.0 %)

Marital status^c^					
Married	2023 (21.0 %)	1462 (34.4 %)	3071 (27.9 %)	47 (11.8 %)	6603 (25.9 %)
Not married	8712 (77.9 %)	3370 (64.3 %)	7840 (70.4 %)	131 (31.6 %)	20,053 (71.8 %)
Missing	369 (1.1 %)	191 (1.3 %)	581 (1.7 %)	1179 (56.6 %)	2320 (2.3 %)

Insurance type^d^					
Medicaid	7086 (56.5 %)	2839 (47.9 %)	5453 (40.0 %)	678 (52.2 %)	16,056 (48.2 %)
Uninsured	1600 (20.0 %)	540 (14.7 %)	1689 (19.3 %)	105 (8.5 %)	3934 (18.7 %)
Other	2418 (23.5 %)	1644 (37.4 %)	4350 (40.6 %)	574 (39.3 %)	8986 (33.2 %)

Employment status^e^					
Employed	3884 (39.8 %)	1776 (41.8 %)	5857 (56.8 %)	170 (121.3 %)	11,687 (46.9 %)
Unemployed	6960 (59.7 %)	3088 (57.3 %)	5204 (42.1 %)	114 (29.2 %)	15,366 (51.5 %)
Missing	260 (0.5 %)	159 (0.9 %)	431 (1.0 %)	1073 (49.5 %)	1923 (1.7 %)

Frequent mental distress^f^					
Yes	2440 (21.1 %)	805 (14.9 %)	1051 (8.5 %)	113 (10.2 %)	4409 (14.7 %)
No	8423 (76.7 %)	4079 (82.3 %)	10,223 (89.7 %)	622 (52.7 %)	23,347 (82.7 %)
Missing	241 (2.2 %)	139 (2.3 %)	218 (1.8 %)	622 (37.1 %)	1220 (2.6 %)

Self-rated health status					
Excellent	711 (7.3 %)	430 (10.5 %)	2001 (19.6 %)	191 (13.7 %)	3333 (13.0 %)
Very good	1856 (19.0 %)	883 (19.2 %)	2926 (28.0 %)	268 (21.6 %)	5933 (22.8 %)
Good	3232 (29.5 %)	1477 (30.1 %)	3351 (28.5 %)	411 (29.0 %)	8471 (29.2 %)
Fair	3540 (30.5 %)	1461 (26.9 %)	2343 (17.8 %)	267 (20.8 %)	7611 (24.5 %)
Poor	1759 (13.6 %)	769 (13.2 %)	870 (6.1 %)	171 (12.4 %)	3569 (10.4 %)
Missing	6 (0.1 %)	3 (0.1 %)	1 (0.0 %)	49 (2.5 %)	59 (0.1 %)

Chronic health condition^g^					
Yes	5209 (41.2 %)	2802 (49.9 %)	4121 (29.4 %)	134 (19.2 %)	12,266 (37.3 %)
No	5508 (55.4 %)	2084 (47.4 %)	6984 (67.5 %)	157 (26.7 %)	14,733 (58.6 %)
Missing	387 (3.4 %)	137 (2.7 %)	387 (3.1 %)	1066 (54.1 %)	1977 (4.1 %)

Rao-Scott chi-square tests compared current, former, and never smokers across variables. All differences were statistically significant at the p < 0.05 level.

a Other race includes those who reported (non-Hispanic) multiple races.

b OMAS defines county types in accordance with guidance from the Appalachian Regional Commission (ARC), US Census Bureau, and the Federal Office of Rural Health Policy at the Health Resources and Services Administration (HRSA).

c Combines unmarried couples, divorced, never married, and widowed.

d Other combines directly purchased insurance, employer-sponsored insurance, and self-rated other insurance plans.

e Participants were asked if they had full- or part-time employment during the last week.

f Frequent mental distress was defined as having at least 14 days in a month where a mental health condition or emotional problem restricted oneself from doing work or other usual activities.

g Defined as having a diagnosed hypertension, heart attack (myocardial infarction), coronary artery disease, congestive heart failure, or diabetes mellitus.

**Table 2:** Weighted distributions of health care access measures among low-income adults by smoking status: Ohio Medicaid Assessment Survey, 2012–2019.

	Current Smokers (N = 11104)	Former smokers(N = 5023)	Never smokers(N = 11492)	Unknown smoking status (N = 1357)
Difficulty paying medical bills	4317 (42.4 %)	1896 (40.7 %)	3525 (31.6 %)	45 (7.9 %)
Unmet other health care needs	1974 (19.0 %)	826 (16.3 %)	1346 (11.6 %)	28 (6.8 %)
Unmet mental, emotional, and counseling care needs	1668 (16.6 %)	532 (11.3 %)	979 (8.6 %)	19 (4.0 %)
Unmet dental care needs	3202 (31.2 %)	1259 (25.8 %)	2331 (20.9 %)	37 (7.2 %)
Having no usual source of care	1071 (11.2 %)	359 (8.4 %)	1221 (12.5 %)	15 (4.2 %)
Unmet dental care needs	3202 (31.2 %)	1259 (25.8 %)	2331 (20.9 %)	37 (7.2 %)

**Fig. 2:**
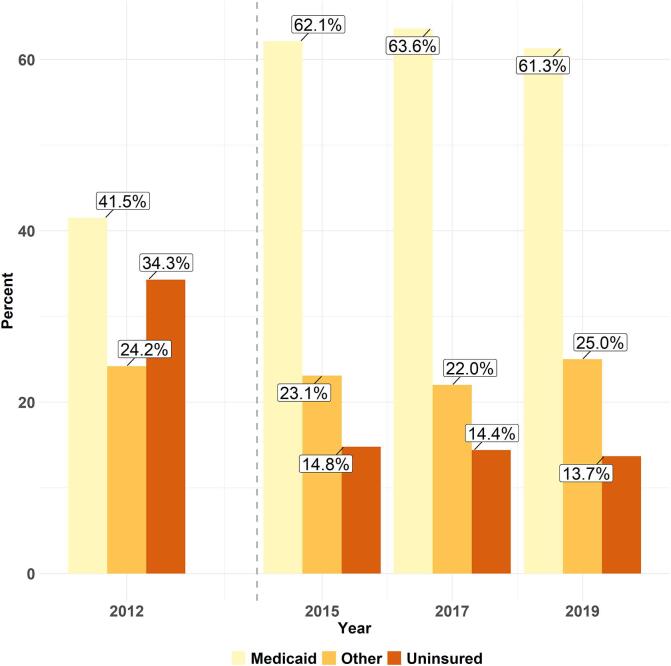
Weighted proportions of adult low-income smokers by insurance type and year: Ohio Medicaid Assessment Survey, 2012–2019. Dashed vertical grey line indicates Medicaid expansion in Ohio in 2014. “Other” insurance combines directly purchased insurance, employer-sponsored insurance, and self-reported other insurance plans.

Regardless of smoking status, access improved among low-income adults between 2012 and 2019. However, most of these changes were not linear, and there were variations in trends among current, former, and never smokers. Fig. 2 shows that more low-income smokers were covered by Medicaid after 2014 and the proportion of uninsured smokers was more than halved. Additionally, access measures among smokers showed disparities by insurance type with uninsured smokers facing the most unmet needs with little improvement across years (Supplementary Fig. S2).

### 3.1 Health care access among current smokers

**Fig. 3:**
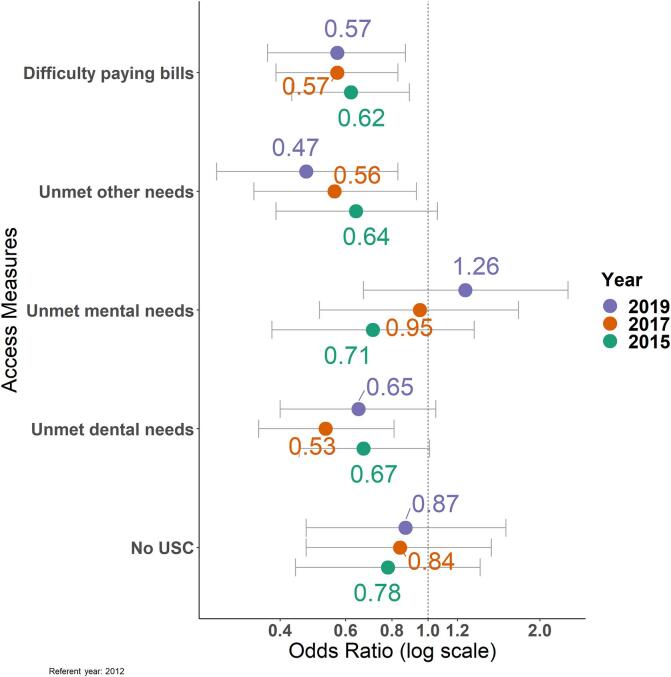
Adjusted odds ratios for health care access measures among low-income adult smokers: Ohio Medicaid Assessment Survey, 2012-2019.^a^ USC = usual source of care ^a^Data were obtained from the Ohio Medicaid Assessment Survey, a survey representative of Ohio’s population pooled across four consecutive cycles in 2012, 2015, 2017, and 2019. All analyses were survey-weighted and adjusted for age, gender, race/ethnicity, county type, educational attainment, and presence of frequent mental distress. Multiple imputation was used to impute missing data due to item nonresponse. Adjusted odds ratios and standard errors across the imputed datasets were obtained using Rubin’s rules (Rubin, 1987).

Improvements in health care access measures were observed for smokers (Fig. 3). Compared to 2012, the odds for unmet dental care needs for current smokers were lower in 2015 (Adjusted Odds Ratio (aOR) = 0.67, 95 % Confidence Interval (CI) = 0.45–1.01), 2017 (aOR = 0.53, 95 % CI = 0.35–0.81), and 2019 (aOR = 0.65, 95 % CI = 0.40–1.05) (p for trend < 0.001), although the differences for 2015 and 2019 (vs 2012) did not quite reach statistical significance. Similarly, compared to 2012, the odds for unmet other health care needs were lower in 2015 (aOR = 0.64, 95 % CI = 0.39–1.06), 2017 (aOR = 0.56, 95 % CI = 0.34–0.93), and 2019 (aOR = 0.47, 95 % CI = 0.27–0.83) (p for trend < 0.001). The odds for difficulty paying medical bills were also significantly lower in 2015 (aOR = 0.62, 95 % CI = 0.43–0.89), 2017 (aOR = 0.57, 95 % CI = 0.39–0.83) and 2019 (aOR = 0.57, 95 % CI = 0.37–0.87) (p for trend < 0.00 l1). Compared to 2012, the odds of having a USC did not statistically improve for current smokers. Estimates also suggested unmet mental, emotional, and counseling care among current smokers increased over time. However, differences across years were not statistically significant.

### 3.2 Health care access among former and never smokers

Similar to current smokers, there was an improvement in unmet other health care needs and difficulty paying medical bills for former smokers (Table 3). However, the most notable and statistically significant improvements were observed for never smokers. Compared to 2012, never smokers experienced significant improvements in having a USC, unmet other health care needs, and difficulty paying medical bills. Like current smokers, however, mental health care needs for never smokers implied an upward trajectory over time (Table 3).

**Table 3:** Adjusted odds ratios for healthcare access measures among low-income adults by smoking status and year: Ohio Medicaid Assessment Survey, 2012-2019a.

Access measure	Year	Current smokers aOR (95 % CI)	p-value for trend	Former smokers aOR (95 % CI)	p-value for trend	Never smokers aOR (95 % CI)	p-value for trend
Difficulty paying medical bills	2012 (Ref)	1.0	<0.001	1.0	<0.001	1.0	<0.001
	2015	**0.62 (0.43**–**0.89)**		0.66 (0.42–1.01)		**0.73 (0.62**–**0.85)**	
	2017	**0.57 (0.39**–**0.83)**		0.65 (0.42–1.02)		**0.70 (0.60**–**0.82)**	
	2019	**0.57 (0.37**–**0.87)**		0.63 (0.38–1.03)		**0.66 (0.55**–**0.79)**	

Unmet other health care needs	2012 (Ref)	1.0	<0.001	1.0	<0.001	1.0	<0.001
	2015	0.64 (0.39–1.06)		0.56 (0.32–1.00)		**0.68 (0.55**–**0.84)**	
	2017	**0.56 (0.34**–**0.93)**		**0.55 (0.31**–**0.99)**		**0.61 (0.49**–**0.75)**	
	2019	**0.47 (0.27**–**0.83)**		**0.44 (0.23**–**0.84)**		**0.51 (0.40**–**0.65)**	

Unmet mental, emotional, and counseling care needs	2012 (Ref)	1.0	0.006	1.0	0.380	1.0	0.004
	2015	0.71 (0.38–1.33)		0.64 (0.30–1.34)		0.82 (0.62–1.09)	
	2017	0.95 (0.51–1.75)		0.63 (0.30–1.31)		1.16 (0.88–1.52)	
	2019	1.26 (0.67–2.38)		1.18 (0.56–2.50)		**1.33 (1.01**–**1.76)**	

Unmet dental care needs	2012 (Ref)	1.0	<0.001	1.0	<0.001	1.0	<0.001
	2015	0.67 (0.45–1.01)		0.76 (0.48–1.23)		0.86 (0.72–1.02)	
	2017	**0.53 (0.35**–**0.81)**		**0.50 (0.30**–**0.82)**		**0.58 (0.49**–**0.70)**	
	2019	0.65 (0.40–1.05)		0.65 (0.37–1.14)		0.74 (0.60–0.91)	

Having no usual source of care	2012 (Ref)	1.0	0.437	1.0	0.179	1.0	0.008
	2015	0.78 (0.44–1.38)		0.69 (0.34–1.43)		**0.72 (0.57**–**0.91)**	
	2017	0.84 (0.47–1.48)		0.82 (0.40–1.69)		**0.73 (0.58**–**0.92)**	
	2019	0.87 (0.47–1.62)		0.70 (0.32–1.53)		**0.69 (0.54**–**0.89)**	

a Data were obtained from the Ohio Medicaid Assessment Survey, a survey representative of Ohio’s population pooled across four consecutive cycles in 2012, 2015, 2017, and 2019. All analyses were survey-weighted and adjusted for age, gender, race/ethnicity, county type, educational attainment, and presence of frequent mental distress. Multiple imputation was used to impute missing data due to item nonresponse. Adjusted odds ratios and standard errors across the imputed datasets were obtained using Rubin’s rules. (Rubin, 1987).

### 3.3 Sensitivity analyses

Results from the main analyses using multiply imputed data were robust to the sensitivity analysis using complete cases with slightly larger standard errors and point estimates that were marginally farther away from the null (Supplementary Table S2).

## 4 Discussion

The main finding of this study is that some, but not all, health care access measures improved for low-income smokers following Ohio’s Medicaid expansion. Specifically, dental care needs, other health care needs (e.g., medical exams or medical supplies), and the ability to pay medical bills improved. There were no meaningful changes in having a USC but there was a suggestion of an upward trend in unmet mental, emotional, or counseling care needs. We also found that changes in access measures differed by smoking status as the most notable progress was among never smokers. We contextualize these findings using the guiding theoretical framework of health care access.

### 4.1 Approachability

To the best of our knowledge, the finding on the lack of improvement in having a USC among low-income smokers is novel. Despite the lack of direct comparisons, our findings align with a previous study that found no association between Medicaid expansion in Ohio and changes in having a USC in other vulnerable groups such as low-income women of reproductive age. (Farietta et al., 2018) Additionally, the improvement observed for never smokers mirrors studies showing a higher likelihood of having a USC among low-income adults following Medicaid expansion. (Sommers et al., 2017, Shartzer et al., 2016) It is generally believed that a health care provider in a primary care setting provides better care than equally qualified but not usual providers as they possess more knowledge of a patient’s history, values, and other attitudes. (Gray et al., 2003) Smokers’ altered risk perceptions, (Weinstein et al., 2005) knowledge about harms of smoking, (Stewart et al., 2013) and lower health insurance literacy (Braun et al., 2017) are all potential explanations for this observed association. It is also possible that perceived provider discrimination among smokers, which is related with low preventive care utilization, (Trivedi and Ayanian, 2006) impacts smokers’ decision for not having a USC. Similarly, although rural smokers have been characterized as having a relatively high trust in their provider, (Nelms et al., 2014) the overall role of medical mistrust in smokers’ decision against having a USC can not be ruled out. Given its implications for health care costs and health disparities, future research should explore potential barriers to having a USC among smokers and forward recommendations for improving the measure. (Office of Disease Prevention and Health Promotion and Department of Health and Human Services, 2022).

### 4.2 Availability and accommodation

The reduction we observed in unmet dental care needs for low-income smokers is supported by other evidence of increased access and utilization of dental care services following Medicaid expansion. (Agency for Healthcare Research and Quality, 2019, Nasseh and Vujicic, 2017) The trend is also attributed to a state’s Medicaid policy. Ohio; for example, is one of only 19 states providing “extensive” dental care coverage, including diagnostic, preventive, and restorative services. (Hinton, 2016) Still; smokers’ unmet dental care needs are higher than non-smokers’, and the decline observed from 2012 to 2017 was not sustained in 2019. Despite the gains in coverage, smokers may still face difficulties finding dental care providers who are Medicaid-contracted. In 2016, only 39 % of providers nationwide and 33 % of providers in Ohio accepted patients on Medicaid or Children’s Health Insurance Plans. (American Dental Association Health Policy Institute, 2022) In addition; lower health insurance literacy among smokers, encompassing limited knowledge about selecting and enrolling in suitable plans, lower comprehension of health benefits, and cost-sharing concepts, is an important determinant of access to care. (Braun et al., 2017).

These findings also emphasize state-level policy revisions to improve dental care access to vulnerable groups such as individuals with diabetes or disabilities (Le and Burroughs, 2019) as well as the expansion of similar initiatives to low-income smokers given they are susceptible to various dental health problems. Unmet other health care needs, closely tied to health care affordability, also declined over time. This is likely attributed to the expansion of benefits under Medicaid expansion. For instance, Ohio expanded coverage for diagnostic, screening, or preventive services (e.g., chest X-rays) and medical equipment (e.g., wheelchairs) with no co-payments. (KFF, 2019a, KFF, 2019b).

Regardless of smoking status, we observed a potential increase in unmet mental, emotional, or counseling care needs over time. Current research suggests little evidence of long-term changes in mental health care access among low-income nonelderly adults following Medicaid expansion. However, there is support for improved access for specific subgroups such as low-income adults with depression (Fry and Sommers, 2018) and psychological distress. (Mojtabai, 2021) In general, the literature on the impact of Medicaid expansion on mental health care utilization among low-income adults is mixed. (KFF, 2020) Nonetheless, our findings reflect stalled mental health care access increases after initial gains seen in 2015, (Robertson-Preidler et al., 2020) with post-expansion changes in utilization remaining relatively consistent with pre-expansion levels for those with existing mental health conditions. (Mojtabai, 2021).

Improved access to mental health improves smoking cessation, (Cook et al., 2014) yet barriers to mental health care access for vulnerable populations remain. (Robertson-Preidler et al., 2020, Medicaid and CHIP Payment, and Access Commission, 2021) For example, the federal repeal of the individual mandate penalty and removal of direct subsidies and cost-sharing payments intended to lower costs for low-income individuals are associated with higher premiums and increases in the uninsured rate. (Tolbert et al., 2020, Semanskee and Claxton, 2017) Furthermore, changes in health care policy threaten to widen existing disparities. For example, Medicaid work requirements, which would require Medicaid recipients to work at least 20 h a week to maintain coverage, were proposed by the Trump administration. (KFF, 2022b) These requirements could have disproportionately impacted smokers, who have a high burden of existing mental care needs and are less likely to meet be eligible. (Robertson-Preidler et al., 2020) Ohio is among the states where such a policy was introduced in 2019, although it was suspended by the Biden administration citing challenges posed by the COVID-19 pandemic.

### 4.3 Affordability

Finally, we found a steady decline in difficulty paying medical bills among smokers. This finding is in line with a study that found low-income smokers in Medicaid expansion states like Ohio had lower cost-related barriers to access to care than low-income smokers in non-expansion states. (Brown et al., 2018) The findings are also consistent with studies showing overall gains in affordability and reductions in avoiding care due to cost following Medicaid expansion. (Griffith et al., 2017) Improvements could also be attributed to enhanced insurance coverage afforded by the ACA including premium tax credits for private insurance, elimination of exclusion based on preexisting conditions, and statement of limits on out-of-pocket costs. Although these findings are highly encouraging, it is critical to assess the impact of the ever-changing policy environment to ensure continuing progress in the affordability of care. Further research is also needed to decipher the role of insurance in the association between smoking and affordability of care and access measures.

### 4.4 Limitations

The first limitation of the study is that findings may not be generalizable or transportable to other states and should be evaluated considering state-by-state variations in Medicaid eligibility, coverage patterns, and other health system characteristics. Studies, however, show little variation in changes in access outcomes across states that underwent Medicaid coverage expansion, (Fry and Sommers, 2018) suggesting these findings may be broadly applicable to these contexts. Second, although our research characterizes trends in access measures over time and the association with smoking, the repeated cross-sectional design does not allow temporal inferences. However, we believe that the notable decline in uninsured low-income smokers is strongly correlated with Medicaid expansion. In relation to this, the role of health insurance status needs further investigation, ideally using data that allows temporal inference. Smoking determines health insurance coverage, (Kaplan et al., 2014) while health insurance directly affects access to care. This suggests that insurance coverage could be a potential mediator of the relationship between smoking and access to care. Third, although we included the main confounders of health care access in our study, we did not account for other unmeasured variables, such as sexual orientation, that could result in residual confounding. Fourth, the measure for difficulty paying medical bills is based on a question assessing affordability of medical costs beyond the individual and could be impacted by factors such as insurance coverage for other family members which was not accounted for in the study. Finally, the guiding conceptual framework (Levesque’s Conceptual Framework of Health Care Access) was not comprehensively covered by survey questions in OMAS, and we could not evaluate some access measures because they were either unavailable or inconsistently measured across years. Attitudes towards health care providers or the health system, ability to reach providers, unmet substance use or alcohol treatment needs, and affordability of prescription drugs are examples of such measures.

## 5 Conclusion

Overall, health care access among low-income adult smokers showed improvement following Ohio’s Medicaid expansion in 2014, although not all access measures improved. Moreover, the progress across most access measures for smokers is not as notable as that of non-smokers, particularly never-smokers. Enduring and upcoming barriers to health care access among smokers, especially amid the COVID-19 pandemic, require attention. Future studies are needed to evaluate the role of changes to federal and state health care policy and explore unique barriers to accessing care among smokers. Research is also needed to assess the impact of COVID-19 and resultant changes in health care access and delivery along with its role in potentially exacerbating existing disparities.

## Declaration of Competing Interest

The authors declare that they have no known competing financial interests or personal relationships that could have appeared to influence the work reported in this paper.

## Data Availability

The data is publicly available and this has been indicated in the manuscript.
