# Supplementary material for: Examining trends in health care access measures among low-income adult smokers in Ohio: 2012–2019
Source: Prev Med Rep. 2023 Jan 2;31:102106. doi: 10.1016/j.pmedr.2022.102106 (PMC9938324; doi:10.1016/j.pmedr.2022.102106)
Supplement: Supplementary data 1 [file mmc1.docx]

## Supplementary Material

Missing data analysis and multiple imputation procedures

Multiple imputation was performed on the full data (i.e., not restricted by income or age) and separately for each survey cycle. The imputation model used all covariates that were used in the analytic model. Additionally, an indicator for income (dichotomous indicator for being at or below 138% FPL vs. above 138% FPL), insurance status (insured vs. uninsured), and chronic health condition were included in the models due to their association with non-response to the exposure and outcome measures.^1,2^ Diagnostic graphs were used to assess model fit and plausibility of generated imputations. Multiple imputation was performed using the *mice* package (version 3.14.0) in R.^3^

Table S1. Percent of missingness in the analytic sample^a^ for mental distress, smoking status, and healthcare access measures.

| Variable | Missing (%) |
| --- | --- |
| Difficulty paying medical bills | 7.2% |
| Unmet other health care needs | 6.8% |
| Unmet mental, emotional, and counseling care needs | 6.7% |
| Unmet dental care needs | 6.6% |
| Usual source of care | 6.5% |
| Smoking status | 4.7% |
| Frequent mental distress | 4.2% |

^a^  N = 28976


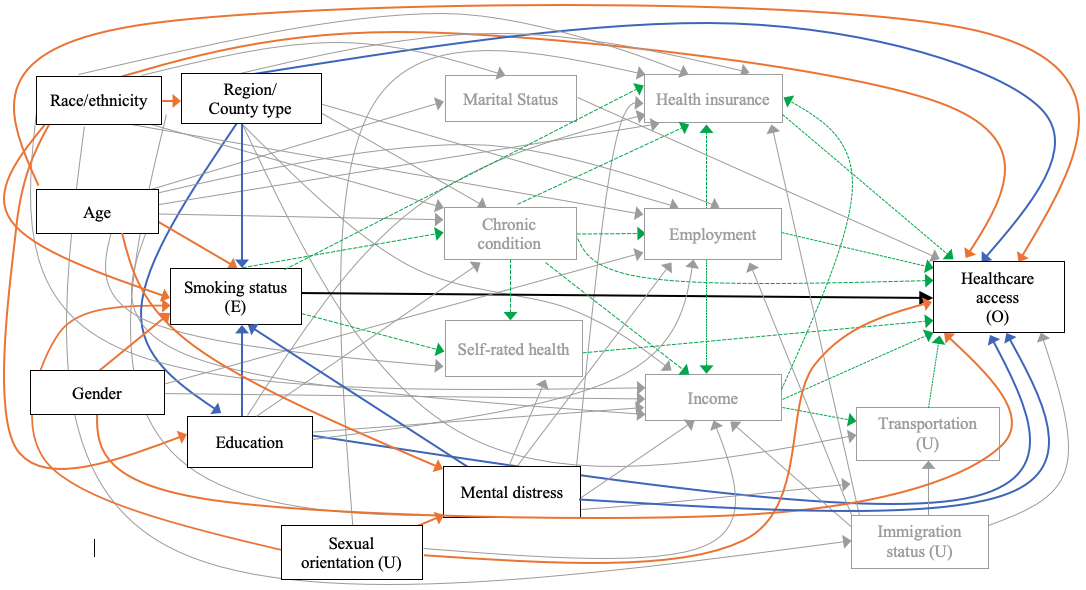


Figure S1. Directed acyclic graph (DAG) depicting relationships between smoking status, healthcare access, and measured and unmeasured variable

E = Exposure; O = Outcome; U = Unmeasured variable

Grayed-out arrows (i.e., arcs) and variables (i.e., nodes) represent paths between smoking status and healthcare access and were not included in the adjusted modes. These variables are either “colliders”^37^ and/or potential mediators of the relationship of interest (e.g., chronic condition). Dashed green lines indicate mediated pathways. Orange and blue lines show adjusted paths from “ancestors” and their “descendants,” respectively. DAG analysis was carried out using the *ggdag* package in R.^72^


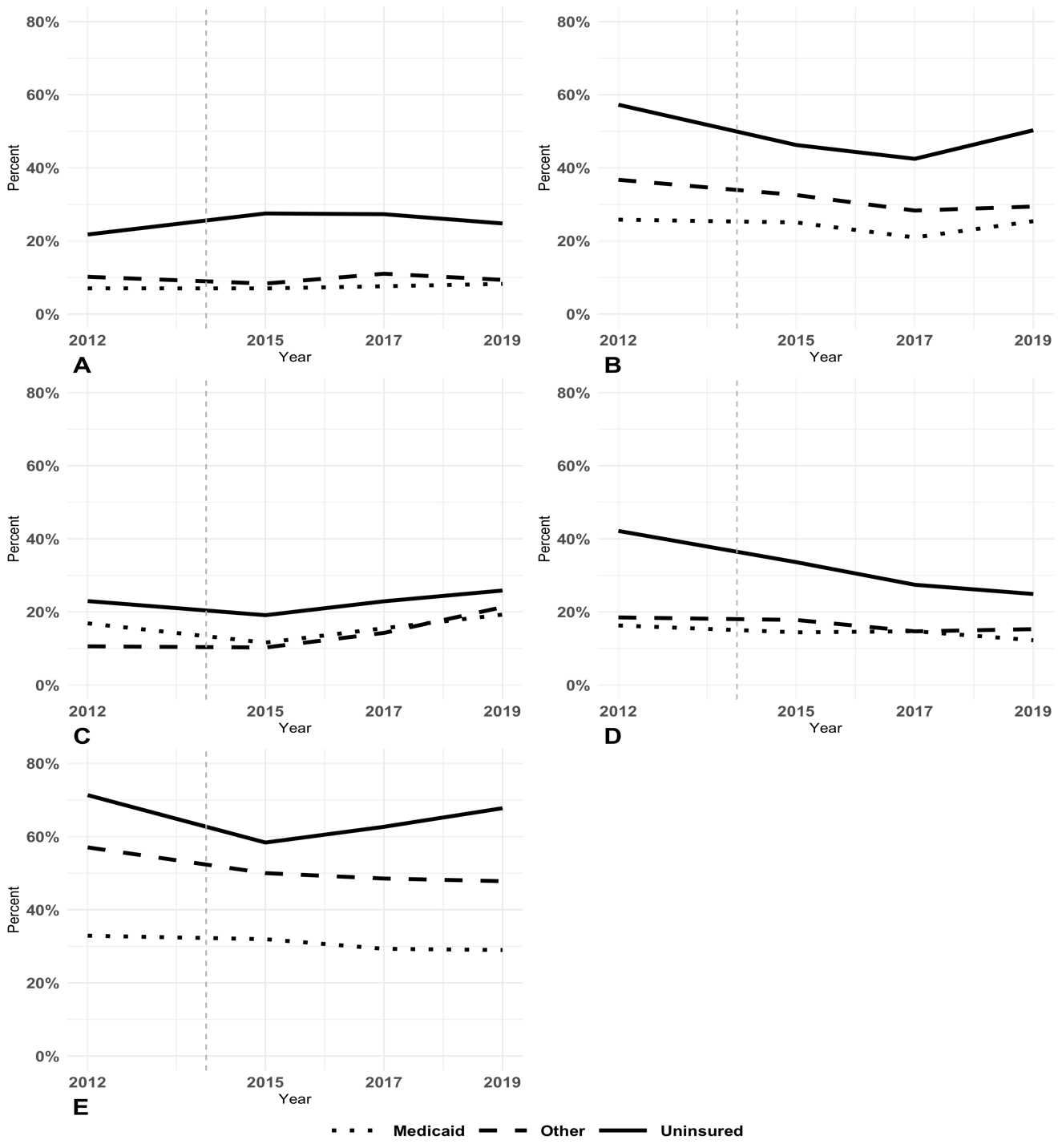


Figure S2. Weighted proportions for healthcare access measures among low-income smokers by insurance type: Ohio Medicaid Assessment Survey, 2012-2019

A: Not having a usual source of care

B: Unmet dental care needs

C: Unmet mental, emotional, or counseling care needs

D: Unmet other (e.g., medical exams or supplies) healthcare needs

E: Difficulty paying medical bills

“Other” combines directly purchased insurance, employer-sponsored insurance, and self-rated other insurance plans.

Dashed vertical grey line indicates Medicaid expansion in Ohio in 2014.

Table S2. Adjusted odds ratios from complete case analysis for healthcare access measures among low-income adults by smoking status and year: Ohio Medicaid Assessment Survey, 2012-2019^a^

| Access measure | Year | Current smokers  aOR (95% CI) | Former smokers  aOR (95% CI) | Never smokers  aOR (95% CI) |
| --- | --- | --- | --- | --- |
| Having no usual source of care | 2012 (Ref) | 1.0 | 1.0 | 1.0 |
|  | 2015 | 0.75 (0.42-1.35) | 0.69 (0.33-1.44) | **0.70 (0.55-0.88)** |
|  | 2017 | 0.81 (0.45-1.44) | 0.84 (0.40-1.74) | **0.72 (0.57-0.91)** |
|  | 2019 | 0.86 (0.46-1.60) | 0.67 (0.30-1.50) | **0.67 (0.52-0.86)** |
| Unmet dental care needs | 2012 (Ref) | 1.0 | 1.0 | 1.0 |
|  | 2015 | 0.66 (0.44-1.00) | 0.76 (0.47-1.22) | 0.85 (0.71-1.01) |
|  | 2017 | **0.53 (0.34-0.81)** | **0.49 (0.29-0.82)** | **0.57 (0.47-0.68)** |
|  | 2019 | 0.63 (0.39-1.03) | 0.65 (0.37-1.14) | 0.73 (0.59-0.90) |
| Unmet mental, emotional, and counseling care needs | 2012 (Ref) | 1.0 | 1.0 | 1.0 |
|  | 2015 | 0.70 (0.37-1.34) | 0.63 (0.29-1.37) | 0.80 (0.60-1.07) |
|  | 2017 | 0.97 (0.52-1.82) | 0.63 (0.30-1.34) | 1.13 (0.86-1.49) |
|  | 2019 | 1.29 (0.67-2.45) | 1.22 (0.56-2.64) | **1.34 (1.01-1.78)** |
| Unmet other healthcare needs | 2012 (Ref) | 1.0 | 1.0 | 1.0 |
|  | 2015 | 0.63 (0.38-1.05) | 0.55 (0.31-1.00) | **0.65 (0.52-0.81)** |
|  | 2017 | **0.56 (0.34-0.94)** | **0.53 (0.29-0.97)** | **0.59 (0.47-0.73)** |
|  | 2019 | **0.46 (0.26-0.82)** | **0.44 (0.23-0.84)** | **0.50 (0.39-0.64)** |
| Difficulty paying medical bills | 2012 (Ref) | 1.0 | 1.0 | 1.0 |
|  | 2015 | **0.60 (0.41-0.87)** | 0.64 (0.41-1.00) | **0.71 (0.61-0.83)** |
|  | 2017 | **0.55 (0.38-0.81)** | **0.63 (0.40-0.99)** | **0.69 (0.59-0.81)** |
|  | 2019 | **0.55 (0.35-0.85)** | 0.62 (0.37-1.02) | **0.65 (0.54-0.78)** |

^a^ Data were obtained from the Ohio Medicaid Assessment Survey, a (roughly) biannual survey representative of Ohio’s population pooled across four consecutive cycles in 2012, 2015, 2017, and 2019. All analyses were survey-weighted and adjusted for age, gender, race/ethnicity, county type, educational attainment, and presence of frequent mental distress.

References

1. van Buuren S. *Flexible Imputation of Missing Data*. Second Edition. Chapman & Hall/CRC Accessed January 30, 2022. https://www.routledge.com/Flexible-Imputation-of-Missing-Data-Second-Edition/Buuren/p/book/9781032178639

2. Hardt J, Herke M, Leonhart R. Auxiliary variables in multiple imputation in regression with missing X: a warning against including too many in small sample research. *BMC Med Res Methodol*. 2012;12(1):184. doi:10.1186/1471-2288-12-184

3. Buuren S van, Groothuis-Oudshoorn K. mice: Multivariate Imputation by Chained Equations in R. *J Stat Softw*. 2011;45:1-67. doi:10.18637/jss.v045.i03
